# *N*-(2,6-Dimethyl­phen­yl)succinamic acid

**DOI:** 10.1107/S1600536809003833

**Published:** 2009-02-06

**Authors:** B. Thimme Gowda, Sabine Foro, B. S. Saraswathi, Hiromitsu Terao, Hartmut Fuess

**Affiliations:** a Department of Chemistry, Mangalore University, Mangalagangotri 574 199, Mangalore, India; b Institute of Materials Science, Darmstadt University of Technology, Petersenstrasse 23, D-64287 Darmstadt, Germany; c Faculty of Integrated Arts and Sciences, Tokushima University, Minamijosanjima-cho, Tokushima 770-8502, Japan

## Abstract

In the amide segment of the title compound, C_12_H_15_NO_3_ {systematic name: 3-[(2,6-dimethyl­phen­yl)amino­carbon­yl]propionic acid}, the N—H and C=O bonds are *anti* to each other. The mol­ecules are packed into a two-dimensional array *via* N—H⋯O and O—H⋯O hydrogen bonds.

## Related literature

For related structures, see: Gowda *et al.* (2007 ▶, 2008 ▶, 2009 ▶).
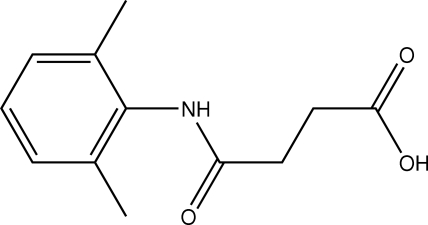


## Experimental

### 

#### Crystal data

C_12_H_15_NO_3_
*M* *_r_* = 221.25
Monoclinic, 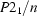

*a* = 7.9633 (8) Å
*b* = 19.889 (2) Å
*c* = 7.9822 (8) Å
β = 111.16 (1)°
*V* = 1179.0 (2) Å^3^
*Z* = 4
Mo *K*α radiation
μ = 0.09 mm^−1^
*T* = 299 (2) K
0.50 × 0.48 × 0.40 mm

#### Data collection

Oxford Diffraction Xcalibur diffractometer with a Sapphire CCD detector
Absorption correction: multi-scan (*CrysAlis RED*; Oxford Diffraction, 2007 ▶) *T*_min_ = 0.958, *T*_max_ = 0.966
6777 measured reflections
2391 independent reflections
1826 reflections with *I* > 2σ(*I*)
*R*_int_ = 0.017

#### Refinement

*R*[*F* ^2^ > 2σ(*F* ^2^)] = 0.043
*wR*(*F* ^2^) = 0.135
*S* = 1.05
2391 reflections
154 parameters
H atoms treated by a mixture of independent and constrained refinement
Δρ_max_ = 0.19 e Å^−3^
Δρ_min_ = −0.17 e Å^−3^

### 

Data collection: *CrysAlis CCD* (Oxford Diffraction, 2004 ▶); cell refinement: *CrysAlis RED* (Oxford Diffraction, 2007 ▶); data reduction: *CrysAlis RED*; program(s) used to solve structure: *SHELXS97* (Sheldrick, 2008 ▶); program(s) used to refine structure: *SHELXL97* (Sheldrick, 2008 ▶); molecular graphics: *PLATON* (Spek, 2003 ▶); software used to prepare material for publication: *SHELXL97*.

## Supplementary Material

Crystal structure: contains datablocks I, global. DOI: 10.1107/S1600536809003833/tk2369sup1.cif

Structure factors: contains datablocks I. DOI: 10.1107/S1600536809003833/tk2369Isup2.hkl

Additional supplementary materials:  crystallographic information; 3D view; checkCIF report

## Figures and Tables

**Table 1:** Hydrogen-bond geometry (Å, °)

*D*—H⋯*A*	*D*—H	H⋯*A*	*D*⋯*A*	*D*—H⋯*A*
N1—H1*N*⋯O1^i^	0.859 (18)	2.059 (19)	2.9120 (16)	171.8 (17)
O2—H2*O*⋯O3^ii^	0.88 (3)	1.79 (3)	2.6686 (18)	178 (3)

Symmetry codes: (i) 
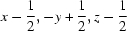
; (ii) 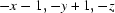.

**Fig. 1.**
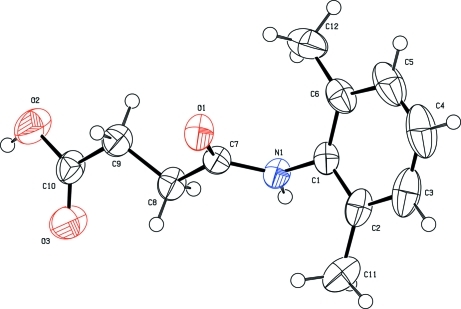
Molecular structure of (I), showing the atom labeling scheme and displacement ellipsoids at the 50% probability level. The H atoms are represented as small spheres of arbitrary radii.

**Fig. 2.**
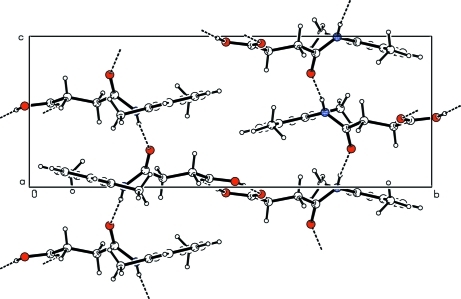
Molecular packing of (I) with hydrogen bonding shown as dashed lines.

## supplementary crystallographic information

### Comment

The amide moiety is an important constituent of many biologically significant compounds. Thus, structural studies of amides are of interest. As a part of an on-going investigation studying the effect of ring and side-chain substitutions on the structures of this class of compounds (Gowda et al., 2007, 2008; 2009), we have determined the crystal structure of N-(2,6-dimethylphenyl)-succinamic acid, (I), with systematic name: 3-[(2,6-dimethylphenyl)-aminocarbonyl]propionic acid, Fig. 1. The N-H and C=O bonds in the amide segment of the structure are anti to each other. The C1-N1-C7-C8, N1-C7-C8-C9, C7-C8-C9-C10 and C8-C9-C10-O2 torsion angles in the side chain are -176.2 (1)°, -145.4 (2)°, -175.5 (1)° and -161.1 (2)°, respectively, and compare to the corresponding values in the structure of i>N-(2-chlorophenyl)-succinamic acid, i.e. 177.5 (2)°, 173.2 (2)°, 178.9 (2)° and 167.7 (2)°, respectively (Gowda et al., 2009).

The presence of N-H···O, between the amide groups, and O-H···O, between the carboxylic acid residues, hydrogen bonding pack molecules into a 2D array (Table 1, Fig.2).

### Experimental

A solution of succinic anhydride (0.025 mole) in toluene (25 ml) was treated dropwise with a solution of 2,6-dimethylaniline (0.025 mole) also in toluene (20 ml) with constant stirring. The resulting mixture was stirred for about 1 h and set aside for an additional hour at room temperature to allow completion of reaction. The mixture was then treated with dilute hydrochloric acid to remove unreacted 2,6-dimethylaniline. The resultant solid was filtered under suction and washed thoroughly with water to remove the unreacted succinic anhydride and succinic acid. The product was recrystallized to constant melting point from ethanol. The purity of the compound was checked by elemental analysis and characterized by its infrared and NMR spectra. The single crystals used in X-ray diffraction studies were grown in ethanolic solution by slow evaporation at room temperature.

### Refinement

The O-bound and N-bound H atoms were located in difference map and their positions refined [O—H = 0.88 (3) Å and N—H = 0.859 (18) Å]. The other H atoms were positioned with idealized geometry using a riding model with C—H = 0.93–0.97 Å, and with *U*_iso_ = 1.2-1.5*U*_eq_(C).

### Crystal data

**Table:**

C_12_H_15_NO_3_	*F*(000) = 472
*M**_r_* = 221.25	*D*_x_ = 1.246 Mg m^−^^3^
Monoclinic, *P*2_1_/*n*	Mo *K*α radiation, λ = 0.71073 Å
Hall symbol: -P 2yn	Cell parameters from 3013 reflections
*a* = 7.9633 (8) Å	θ = 2.7–28.0°
*b* = 19.889 (2) Å	µ = 0.09 mm^−^^1^
*c* = 7.9822 (8) Å	*T* = 299 K
β = 111.16 (1)°	Prism, colourless
*V* = 1179.0 (2) Å^3^	0.50 × 0.48 × 0.40 mm
*Z* = 4	

### Data collection

**Table:**

Oxford Diffraction Xcalibur diffractometer with a Sapphire CCD detector	2391 independent reflections
Radiation source: fine-focus sealed tube	1826 reflections with *I* > 2σ(*I*)
graphite	*R*_int_ = 0.017
Rotation method data acquisition using ω and φ scans	θ_max_ = 26.4°, θ_min_ = 2.9°
Absorption correction: multi-scan (*CrysAlis RED*; Oxford Diffraction, 2007)	*h* = −9→9
*T*_min_ = 0.958, *T*_max_ = 0.966	*k* = −24→24
6777 measured reflections	*l* = −8→9

### Refinement

**Table:**

Refinement on *F*^2^	Secondary atom site location: difference Fourier map
Least-squares matrix: full	Hydrogen site location: inferred from neighbouring sites
*R*[*F*^2^ > 2σ(*F*^2^)] = 0.043	H atoms treated by a mixture of independent and constrained refinement
*wR*(*F*^2^) = 0.135	*w* = 1/[σ^2^(*F*_o_^2^) + (0.0691*P*)^2^ + 0.3248*P*] where *P* = (*F*_o_^2^ + 2*F*_c_^2^)/3
*S* = 1.05	(Δ/σ)_max_ = 0.03
2391 reflections	Δρ_max_ = 0.19 e Å^−^^3^
154 parameters	Δρ_min_ = −0.17 e Å^−^^3^
0 restraints	Extinction correction: *SHELXL97* (Sheldrick, 2008), Fc^*^=kFc[1+0.001xFc^2^λ^3^/sin(2θ)]^-1/4^
Primary atom site location: structure-invariant direct methods	Extinction coefficient: 0.017 (4)

### Special details

**Table:**

Experimental. CrysAlis RED, Oxford Diffraction Ltd., 2007 Empirical absorption correction using spherical harmonics, implemented in SCALE3 ABSPACK scaling algorithm.
Geometry. All e.s.d.'s (except the e.s.d. in the dihedral angle between two l.s. planes) are estimated using the full covariance matrix. The cell e.s.d.'s are taken into account individually in the estimation of e.s.d.'s in distances, angles and torsion angles; correlations between e.s.d.'s in cell parameters are only used when they are defined by crystal symmetry. An approximate (isotropic) treatment of cell e.s.d.'s is used for estimating e.s.d.'s involving l.s. planes.
Refinement. Refinement of *F*^2^ against ALL reflections. The weighted *R*-factor *wR* and goodness of fit *S* are based on *F*^2^, conventional *R*-factors *R* are based on *F*, with *F* set to zero for negative *F*^2^. The threshold expression of *F*^2^ > σ(*F*^2^) is used only for calculating *R*-factors(gt) *etc*. and is not relevant to the choice of reflections for refinement. *R*-factors based on *F*^2^ are statistically about twice as large as those based on *F*, and *R*- factors based on ALL data will be even larger.

### Fractional atomic coordinates and isotropic or equivalent isotropic displacement parameters (Å^2^)

**Table:**

	*x*	*y*	*z*	*U*_iso_*/*U*_eq_	
C1	0.12966 (19)	0.18528 (8)	0.03678 (19)	0.0379 (4)	
C2	0.0916 (2)	0.11993 (8)	0.0772 (2)	0.0480 (4)	
C3	0.2273 (3)	0.07205 (10)	0.1098 (3)	0.0688 (6)	
H3	0.2062	0.0282	0.1376	0.083*	
C4	0.3924 (3)	0.08854 (14)	0.1016 (3)	0.0799 (8)	
H4	0.4816	0.0559	0.1241	0.096*	
C5	0.4251 (3)	0.15276 (13)	0.0604 (3)	0.0693 (6)	
H5	0.5369	0.1632	0.0552	0.083*	
C6	0.2949 (2)	0.20310 (10)	0.0260 (2)	0.0483 (4)	
C7	0.00487 (18)	0.28812 (7)	0.11169 (19)	0.0353 (3)	
C8	−0.1610 (2)	0.33204 (8)	0.0593 (2)	0.0434 (4)	
H8A	−0.2293	0.3264	−0.0679	0.052*	
H8B	−0.2363	0.3174	0.1245	0.052*	
C9	−0.1168 (2)	0.40548 (8)	0.0976 (2)	0.0467 (4)	
H9A	−0.0409	0.4109	0.2229	0.056*	
H9B	−0.0499	0.4212	0.0250	0.056*	
C10	−0.2830 (2)	0.44735 (8)	0.0582 (2)	0.0473 (4)	
C11	−0.0877 (3)	0.10257 (10)	0.0884 (3)	0.0637 (5)	
H11A	−0.1803	0.1074	−0.0280	0.076*	
H11B	−0.1120	0.1323	0.1717	0.076*	
H11C	−0.0854	0.0570	0.1287	0.076*	
C12	0.3334 (3)	0.27264 (12)	−0.0218 (3)	0.0662 (6)	
H12A	0.3733	0.3001	0.0843	0.079*	
H12B	0.2258	0.2916	−0.1077	0.079*	
H12C	0.4256	0.2709	−0.0727	0.079*	
N1	−0.00837 (16)	0.23498 (6)	0.00415 (17)	0.0368 (3)	
H1N	−0.111 (2)	0.2276 (9)	−0.079 (2)	0.044*	
O1	0.13819 (13)	0.30009 (6)	0.24524 (15)	0.0469 (3)	
O2	−0.25948 (19)	0.51129 (6)	0.0379 (2)	0.0750 (5)	
H2O	−0.363 (4)	0.5325 (13)	0.012 (3)	0.090*	
O3	−0.42763 (16)	0.42371 (6)	0.0464 (2)	0.0714 (5)	

### Atomic displacement parameters (Å^2^)

**Table:**

	*U*^11^	*U*^22^	*U*^33^	*U*^12^	*U*^13^	*U*^23^
C1	0.0333 (7)	0.0407 (8)	0.0329 (7)	0.0075 (6)	0.0038 (6)	−0.0065 (6)
C2	0.0541 (10)	0.0393 (9)	0.0399 (9)	0.0074 (7)	0.0042 (7)	−0.0068 (7)
C3	0.0865 (16)	0.0462 (11)	0.0573 (12)	0.0256 (10)	0.0061 (10)	−0.0063 (8)
C4	0.0731 (15)	0.0875 (17)	0.0666 (13)	0.0504 (13)	0.0101 (11)	−0.0078 (12)
C5	0.0444 (10)	0.0996 (18)	0.0593 (12)	0.0256 (11)	0.0134 (9)	−0.0131 (11)
C6	0.0358 (8)	0.0675 (11)	0.0385 (8)	0.0078 (7)	0.0096 (7)	−0.0073 (8)
C7	0.0270 (7)	0.0371 (8)	0.0365 (8)	0.0034 (6)	0.0052 (6)	0.0009 (6)
C8	0.0307 (7)	0.0399 (8)	0.0491 (9)	0.0082 (6)	0.0018 (6)	−0.0039 (7)
C9	0.0341 (8)	0.0415 (9)	0.0577 (10)	0.0060 (6)	0.0084 (7)	−0.0013 (7)
C10	0.0385 (9)	0.0376 (8)	0.0576 (10)	0.0057 (7)	0.0075 (7)	−0.0053 (7)
C11	0.0671 (12)	0.0449 (10)	0.0697 (13)	−0.0105 (9)	0.0132 (10)	0.0004 (9)
C12	0.0486 (10)	0.0862 (15)	0.0702 (13)	−0.0092 (10)	0.0291 (10)	0.0006 (11)
N1	0.0258 (6)	0.0367 (7)	0.0384 (7)	0.0032 (5)	0.0001 (5)	−0.0047 (5)
O1	0.0325 (6)	0.0498 (7)	0.0436 (6)	0.0089 (5)	−0.0042 (5)	−0.0094 (5)
O2	0.0473 (8)	0.0376 (7)	0.1347 (15)	0.0076 (6)	0.0263 (8)	−0.0008 (8)
O3	0.0415 (7)	0.0434 (7)	0.1276 (13)	0.0085 (5)	0.0284 (8)	0.0044 (7)

### Geometric parameters (Å, °)

**Table:**

C1—C6	1.395 (2)	C8—H8A	0.9700
C1—C2	1.398 (2)	C8—H8B	0.9700
C1—N1	1.4306 (18)	C9—C10	1.498 (2)
C2—C3	1.393 (2)	C9—H9A	0.9700
C2—C11	1.503 (3)	C9—H9B	0.9700
C3—C4	1.379 (3)	C10—O3	1.216 (2)
C3—H3	0.9300	C10—O2	1.304 (2)
C4—C5	1.367 (4)	C11—H11A	0.9600
C4—H4	0.9300	C11—H11B	0.9600
C5—C6	1.395 (3)	C11—H11C	0.9600
C5—H5	0.9300	C12—H12A	0.9600
C6—C12	1.495 (3)	C12—H12B	0.9600
C7—O1	1.2260 (17)	C12—H12C	0.9600
C7—N1	1.3415 (19)	N1—H1N	0.859 (18)
C7—C8	1.5115 (19)	O2—H2O	0.88 (3)
C8—C9	1.508 (2)		
			
C6—C1—C2	122.55 (15)	H8A—C8—H8B	107.8
C6—C1—N1	119.54 (15)	C10—C9—C8	111.83 (13)
C2—C1—N1	117.91 (14)	C10—C9—H9A	109.3
C3—C2—C1	117.43 (18)	C8—C9—H9A	109.3
C3—C2—C11	121.43 (18)	C10—C9—H9B	109.3
C1—C2—C11	121.12 (15)	C8—C9—H9B	109.3
C4—C3—C2	121.1 (2)	H9A—C9—H9B	107.9
C4—C3—H3	119.4	O3—C10—O2	122.82 (15)
C2—C3—H3	119.4	O3—C10—C9	122.82 (15)
C5—C4—C3	120.05 (18)	O2—C10—C9	114.37 (15)
C5—C4—H4	120.0	C2—C11—H11A	109.5
C3—C4—H4	120.0	C2—C11—H11B	109.5
C4—C5—C6	121.7 (2)	H11A—C11—H11B	109.5
C4—C5—H5	119.1	C2—C11—H11C	109.5
C6—C5—H5	119.1	H11A—C11—H11C	109.5
C1—C6—C5	117.12 (19)	H11B—C11—H11C	109.5
C1—C6—C12	122.35 (15)	C6—C12—H12A	109.5
C5—C6—C12	120.53 (18)	C6—C12—H12B	109.5
O1—C7—N1	123.55 (13)	H12A—C12—H12B	109.5
O1—C7—C8	121.63 (13)	C6—C12—H12C	109.5
N1—C7—C8	114.79 (12)	H12A—C12—H12C	109.5
C9—C8—C7	112.76 (12)	H12B—C12—H12C	109.5
C9—C8—H8A	109.0	C7—N1—C1	123.37 (12)
C7—C8—H8A	109.0	C7—N1—H1N	117.7 (12)
C9—C8—H8B	109.0	C1—N1—H1N	118.3 (12)
C7—C8—H8B	109.0	C10—O2—H2O	109.4 (16)
			
C6—C1—C2—C3	0.9 (2)	C4—C5—C6—C1	0.5 (3)
N1—C1—C2—C3	−179.61 (14)	C4—C5—C6—C12	−178.95 (19)
C6—C1—C2—C11	179.75 (15)	O1—C7—C8—C9	36.3 (2)
N1—C1—C2—C11	−0.8 (2)	N1—C7—C8—C9	−145.43 (15)
C1—C2—C3—C4	−0.3 (3)	C7—C8—C9—C10	−175.54 (14)
C11—C2—C3—C4	−179.12 (18)	C8—C9—C10—O3	19.1 (3)
C2—C3—C4—C5	−0.2 (3)	C8—C9—C10—O2	−161.05 (17)
C3—C4—C5—C6	0.1 (3)	O1—C7—N1—C1	2.0 (2)
C2—C1—C6—C5	−1.0 (2)	C8—C7—N1—C1	−176.22 (14)
N1—C1—C6—C5	179.54 (14)	C6—C1—N1—C7	−66.5 (2)
C2—C1—C6—C12	178.40 (16)	C2—C1—N1—C7	114.05 (17)
N1—C1—C6—C12	−1.0 (2)		

### Hydrogen-bond geometry (Å, °)

**Table:**

*D*—H···*A*	*D*—H	H···*A*	*D*···*A*	*D*—H···*A*
N1—H1N···O1^i^	0.859 (18)	2.059 (19)	2.9120 (16)	171.8 (17)
O2—H2O···O3^ii^	0.88 (3)	1.79 (3)	2.6686 (18)	178 (3)

Symmetry codes: (i) *x*−1/2, −*y*+1/2, *z*−1/2; (ii) −*x*−1, −*y*+1, −*z*.
